# Supplementary material for: Sigma-1 Receptor Activation Improves Oligodendrogenesis and Promotes White-Matter Integrity after Stroke in Mice with Diabetic Mellitus
Source: Molecules. 2023 Jan 2;28(1):390. doi: 10.3390/molecules28010390 (PMC9823930; doi:10.3390/molecules28010390)
Supplement: Supplementary file 1 [file molecules-28-00390-s001.zip › molecules-2039931-supplementary.pdf]

Sigma-1 Receptor Activation Improves Oligodendrogenesis and Promotes White-Matter Integrity after Stroke in Mice with Diabetic Mellitus

Wenjing Song, Yang Yao, Heling Zhang, Xin Hao, Liping Zhou, Zhixiao Song, Tiantian Wei, Tianyan Chi, Peng Liu *, Xuefei Ji * and Libo Zou *

Department of Pharmacology, Shenyang Pharmaceutical University, 103 Wenhua Road, Shenhe District, Shenyang 110016, China

Supplementary Materials

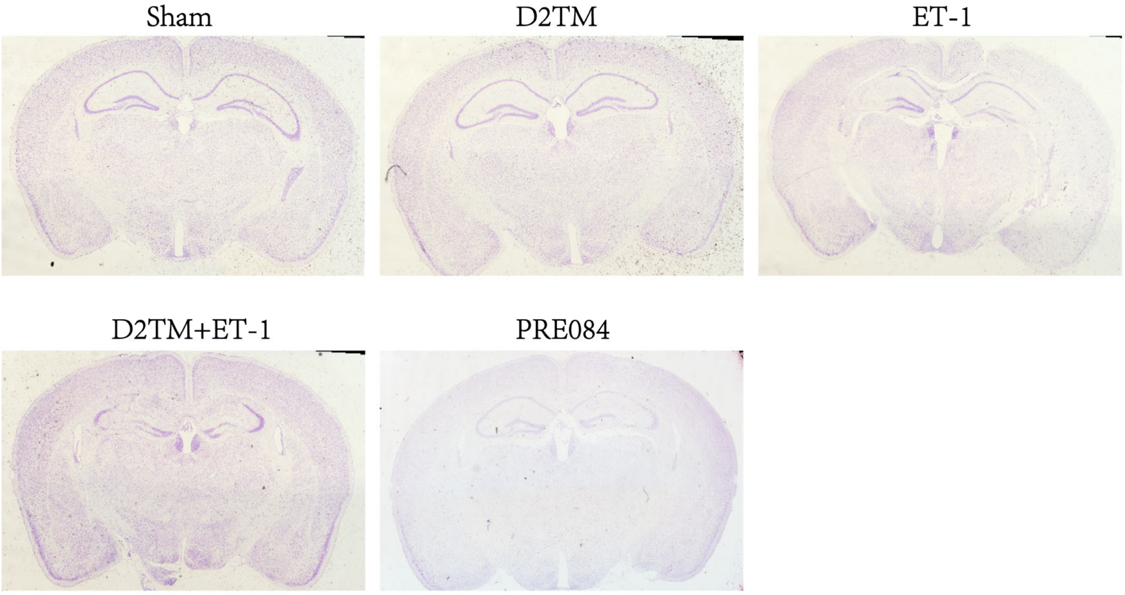

Figure S1. ET-1 injection-induced infarct lesions were tested with Nissl staining. n = 4–5/group.

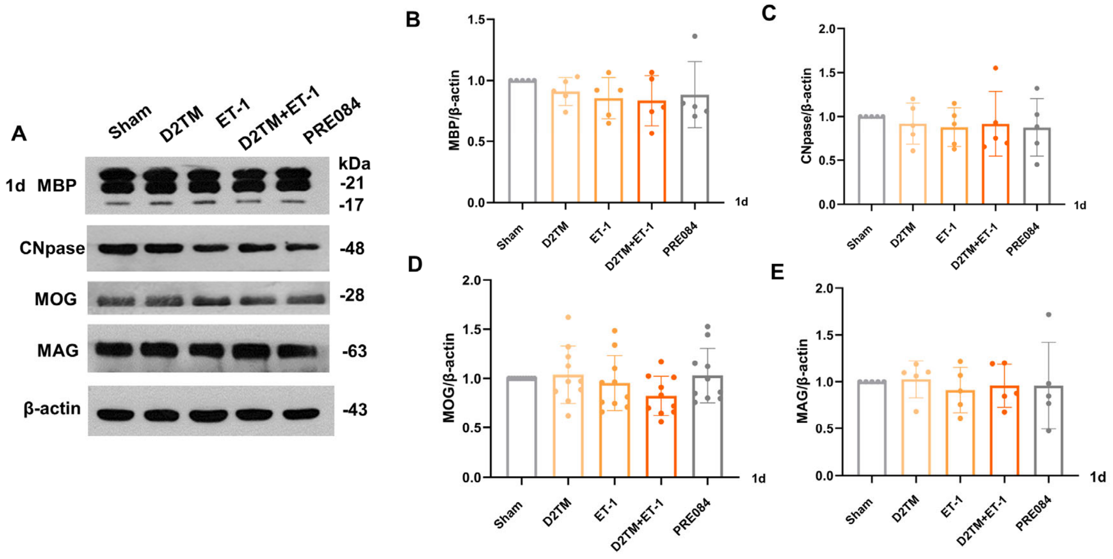

Figure S2. PRE084 promoted mature oligodendrogenesis and myelin regeneration in diabetic mice with stroke at 1 d after ET-1 injection. A) Immunoblot analysis of hippocampal tissue with antibodies against MBP, CNpase, MOG, and MAG. B–E) Quantification of the band densities of MBP, CNpase, MOG, and MAG. Data are expressed as mean ± SEM; $n = 5$/group.
